# O.2.3-5 Workplace Active Certification: the creation of a certification process that values and rewards professional organisations implementing physical activity for their employees

**DOI:** 10.1093/eurpub/ckad133.131

**Published:** 2023-09-11

**Authors:** Frank Vandaele, Adam Evans, Stefano De Dominicis, Michael Gross, Gurvan Heuzé, Ine De Clerck

**Affiliations:** Artevelde University of Applied Sciences, Belgium; University of Copenhagen, Denmark; University of Copenhagen, Denmark; Evaleo, Switzerland; European Federation for Company Sport, France; frank.vandaele@arteveldehs.be; Artevelde University of Applied Sciences, Belgium

## Abstract

**Purpose:**

The workplace is often highlighted as a potential location for promoting physical activity and healthy lifestyles. However, embedding physical activity in the workplace seems less obvious. Due to the unique culture and structure of companies, differences in interpersonal relationships and in personal motivation, as well as local/national differences, it is impossible to offer a simple “one size fits all” strategy that can be applied everywhere in Europe. The 'Workplace Active Certification' (WAC, Erasmus+) project aimed to develop an evidence-based certification process through which companies that successfully embed HEPA in the workplace can be certified as 'physically active'

**Methods:**

To achieve this goal, the programme integrated different types of evidence from different sources. These included a scoping review, multi-stage online interviews with 'pioneer' employers (28) in several (7) European countries, and iterative consultations with cross-sectoral experts in health and physical activity promotion (44). The full certification/audit process was tested and evaluated with 16 pioneer companies. The project included 10 cross-sectoral supporting partners.

**Results:**

All this shared information has resulted in a toolbox with an introductory questionnaire and a comprehensive list of 40 WAC criteria that should be met by any organisation wishing to develop its 'Active Workplace' approach and become 'WAC certified'. The criteria are divided into 5 categories: needs assessment, leadership, planned actions, work environment and evaluation. In 2022, a total of 13 companies were awarded the WAC certificate.

**Conclusions:**

We have developed a certification system with the following characteristics: multi-level, contextualised, targeted, action-based and impact and sustainability oriented. Going through the certification process helps companies to improve their workplace HEPA policies. Achieving certification recognises and rewards professional organisations for implementing HEPA for their employees.

**Funding Source:**

ERASMUS+ European Commission.

